# Research hotspots and trend of wrist arthroscopy: A bibliometrics analysis from 2013 to 2023

**DOI:** 10.1097/MD.0000000000037684

**Published:** 2024-04-05

**Authors:** Chengyin Lu, Zhiqiang Luo, Li Zeng, Zehua Rao, Mingxuan Wang, Xiaohui Wang, Hui Xiong, Biao Zhou

**Affiliations:** a Department of the Second Clinic College of Chinese Medicine, Hunan University of Chinese Medicine, Changsha, China; b Department of Orthopedics, Luoyang Orthopedic-Traumatological Hospital of Henan Province (Henan Provincial Orthopedic Hospital), Luoyang, China; c Department of the second Clinical Medical College, Shanxi Medical University, Taiyuan, China; d Department of Orthopedics, The First People’s Hospital of Xiangtan City, Xiangtan, China; e Department of Orthopedics, Wangjing Hospital of Chinese Academy of Chinese Medical Science, Beijing, China.

**Keywords:** bibliometrics, CiteSpace, R package “Bibliometrix,”, VOSviewers, wrist arthroscopy

## Abstract

**Background:**

Wrist arthroscopy technology is a surgical technology invented in recent years and widely used in clinical treatment of various wrist diseases. This study uses the methods of bibliometrics and visual analysis to understand the global research status, research hotspots, and future development trends of wrist arthroscopy.

**Methods:**

The relevant literature of global publications on wrist arthroscopy from 2013 to 2023 was extracted from the Web of Science Core Collection database, and the annual output, cooperation, hot spots, research status, and development trend of this field were analyzed by using the bibliometric software (VOSviewers, CiteSpace, and the R package “Bibliometrix”).

**Results:**

A total of 635 articles were included, from 2013 to 2023, the number of publications related to wrist arthroscopy showed an overall upward trend, the USA, France, and China are the top 3 countries in terms of the number of publications, whereas Mayo Clinic is the institution with the highest number of publications, Ho PC holds a core position in this field, keyword analysis indicates that the research hotspots are the applications of wrist arthroscopy in triangular fibrocartilage complex injuries, scaphoid nonunion, and avascular necrosis of the lunate.

**Conclusion subsections:**

Wrist arthroscopy has shown tremendous potential in treating various wrist diseases. However, there are still some challenges in its research domain. With continuous deep research, strengthened international collaboration, and ongoing technological advancements, wrist arthroscopy has the potential to become the standard treatment in hand surgery, offering more efficient and safer treatment options for patients worldwide.

## 1. Introduction

Wrist arthroscopy is a novel technique used for diagnosing and treating wrist joint injuries and conditions.^[1]^ In this procedure, a mini high-definition camera through a small incision in the wrist, providing visualization that closely resembles traditional open joint surgery.^[2]^ This technique allows for thorough assessment and examination of intra-articular lesions within the wrist joint. Moreover, repairs of these structures can be performed through several small incisions, thereby minimizing tissue damage compared to conventional open surgery. Wrist arthroscopy offers several advantages over open surgery, such as smaller and more aesthetically pleasing incisions, as well as the avoidance of damage to surrounding ligaments and joint capsule during exposure.^[3]^ Furthermore, it carries a reduced risk of noticeable surgical scars. Consequently, patients experience less postoperative pain, shorter hospital stays, and faster recovery times. Importantly, the minimal disruption to the joint capsule and ligaments during surgery also reduces the formation of postoperative adhesions, facilitating optimal joint function, and recovery.^[4]^

Initially developed and primarily used for diagnostic purposes, wrist arthroscopy has gradually evolved into a valuable adjunctive treatment modality for various wrist joint injuries and conditions.^[5,6]^ Over the past decade, it has become a standard procedure for certain wrist joint injuries and conditions. In theory, except for a few procedures like wrist joint arthroplasty, most wrist joint injuries and conditions can be treated using minimally invasive techniques with the assistance of wrist arthroscopy.^[7]^ With increasing patient demand for minimally invasive surgery and the ongoing evolution of surgical techniques, the indications for wrist arthroscopy are rapidly expanding. Currently, wrist arthroscopy can be utilized in the clinical treatment of diseases including joint motion injuries, ligament injuries, triangular fibrocartilage complex (TFCC) injuries, dislocations of the distal ulna, scaphoid fractures and nonunions, distal radius fractures, and ulnar impaction syndrome.^[8–11]^ In summary, wrist arthroscopy has revolutionized the diagnosis and treatment of wrist joint pathologies. It offers numerous advantages over traditional open surgery, including smaller incisions, improved aesthetics, reduced postoperative pain, shorter hospital stays, and faster recovery.^[12]^ As indications broaden and advancements continue, wrist arthroscopy continues to reshape the landscape of wrist joint interventions.

Bibliometrics, as a quantitative and visual method of literature analysis, plays a crucial role in scientific research and academia.^[13,14]^ It can examine the output and status of literature in various research topics and is widely used to evaluate research results, identify emerging trends, and evaluate the impact of academic literature. It is now widely used in medical fields, such as orthopedics, autoimmune diseases, etc.^[15–17]^ However, no bibliometric studies on wrist arthroscopy have been found in recent years. Therefore, in this study, we utilized sophisticated bibliometric tools, including CiteSpace,^[18]^ VOSviewer,^[19]^ R package“Bibliometrix,”^[20]^ and an online analysis platform (https://bibliometric.com), to obtain comprehensive data on references related to wrist arthroscopy literature. This encompassed information on countries, authors, institutions, journals, keywords, and research fields. Subsequently, we visualized the data to outline the research hotspots, application trends, and collaborative achievements in the field of wrist arthroscopy. This endeavor aims to enhance our understanding of its role in the management of wrist joint conditions and provide valuable insights and guidance for future research focus.

## 2. Materials and methods

### 2.1. Data sources and search strategies

**Figure 1.**
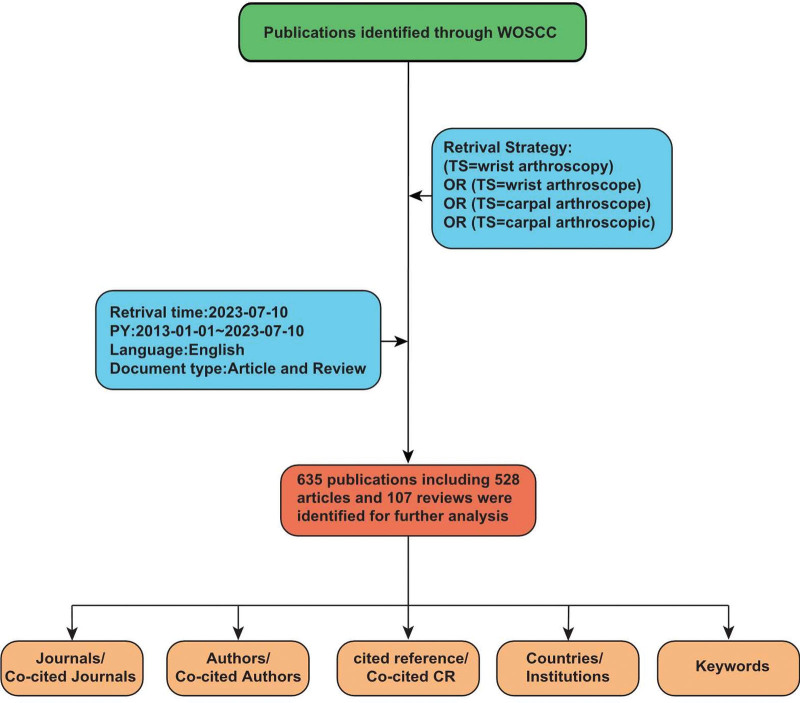
Flowchart of literature identification and analysis process. CR = cited reference, PY = year published, TS = topic, WOSCC = Web of Science Core Collection.

We performed a comprehensive literature search in the Web of Science Core Collection (https://www.webofscience.com/wos/woscc), based on daily updates to the database that may have affected the included studies, the literature search was completed within a single day (July 10, 2023). The search terms is as follows: ([TS = wrist arthroscopy], OR [TS = wrist arthroscope], OR [TS = carpal arthroscope], OR [TS = carpal arthroscopic]), the type of literature was restricted to “articles” and “reviews,” and the publication period in this study was set from January 1, 2013 to July 10, 2023. Language restricted to English. After the initial search, we screened the titles and abstracts to confirm the eligibility of the article based on predefined inclusion and exclusion criteria. The flowchart of the screening process is shown in Figure 1. For this bibliometric analysis, ethical approval was not necessary as it did not involve direct human or animal subjects, relying solely on public data and literature.

### 2.2. Bibliometric analysis

After the completion of data collection, we extracted relevant bibliometric information from the selected publications, including annual publication count, countries, affiliations, journals, authors, citation status, keywords, etc. To conduct quantitative analysis and visualization of the collected data, we utilized 4 tools: CiteSpace (Version 6.2. R4), VOSviewer (Version 1.6.19), R package “Bibliometrix” (Version 4.0.0) (https://www.bibliometrix.org), and an online analysis platform (https://bibliometric.com).

## 3. Results

### 3.1. Annual publication trends

**Figure 2.**
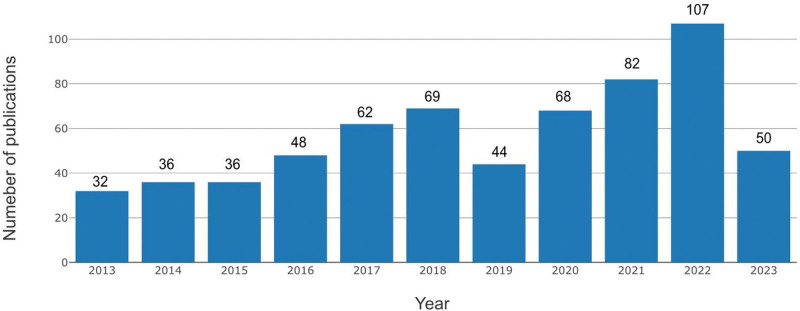
Annual output of arthroscopy publications.

Based on the search conditions and process shown in Figure 1, we collected a total of 635 relevant publications, including 528 articles and 107 reviews. Figure 2 shows the number of publications released each year. It increased from 32 in 2013 to 69 in 2018, demonstrating a continuous upward trend. However, in 2019, the number of publications suddenly decreased to 44, followed by a gradual increase again, reaching a total of 107 publications by 2022. Since 2023 has not concluded, the publication count for that year is insufficient to portray a comprehensive trend.

Regarding the annual number of wrist arthroscopy publications from 2013 to 2023, we consider that it might be influenced by several factors. First, the overall trend of increasing publication numbers from 2013 to 2023 may be attributed to the growing interest of hand surgeons in wrist arthroscopy technology, advancements in wrist arthroscopy, and its expanded clinical applications. During this period, there might have been increased funding opportunities, conferences, and symposiums dedicated to this field, thereby increasing the number of publications annually. For the sudden reduction in the number of publications in 2019, we think there could be several potential reasons. First, academic and research fields often go through cycles of innovation and consolidation. If significant technological advancements were made and published in the years before 2019, researchers might have shifted their focus toward consolidating research results and exploring practical clinical applications instead of producing a large volume of new publications. Therefore, as wrist arthroscopy technology became more widely adopted in clinical practice, the research focus might have shifted from exploratory investigations to more comprehensive and rigorous validation studies. These clinical applications and validation studies often require additional time and effort to collect high-quality patient cohort data, thus potentially leading to a decrease in the number of papers published in 2019. Additionally, the corona virus disease 2019 pandemic that started in 2019 might have had an impact on research output and priorities, such as laboratory closures, reduced funding, and reallocating resources to corona virus disease 2019-related research, could have led to a temporary decline in the number of publications. Nevertheless, based on the overall trend of publication numbers over the years, it is evident that the related publications on wrist arthroscopy are continuously increasing, suggesting significant potential in wrist arthroscopy. Therefore, the research trend of wrist arthroscopy is expected to persist.

### 3.2. Analysis of countries/regions

**Figure 3.**
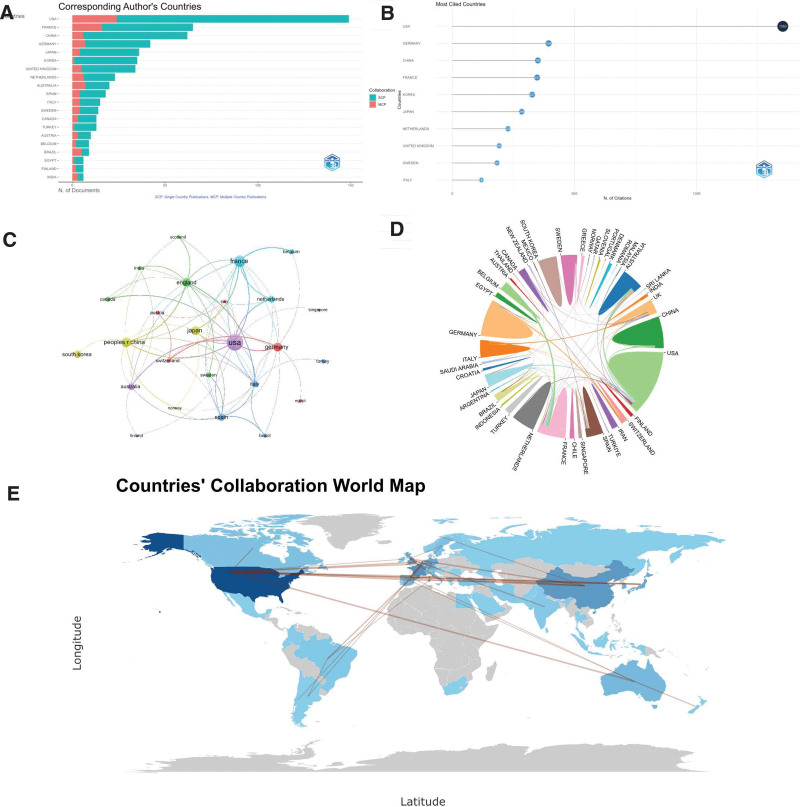
Country distribution of wrist arthroscopy publications. (A) Top 20 countries that produced the largest number of literature. (B) Top 10 most cited countries. (C) The overlay visualization map of country co-authorship analysis. (D) The international cooperation analysis among different countries. (E) The countries’ collaboration world map. MCP = multiple country publications, SCP = single country publications.

We measure the output of countries/regions by calculating the “Corresponding author’s countries.” According to the results shown in Figure 3A and Table 1, among all 635 publications, the USA (N = 149, 23.5%), France (N = 65, 10.2%), and China (N = 62, 9.8%) are the top 3 countries in terms of the number of publications. Their publication count is significantly higher than that of other countries/regions, each having over 60 publications. Following closely are Germany (N = 42, 6.6%), Japan (N = 36, 5.7%), South Korea (N = 35, 5.5%), and the United Kingdom (N = 34, 5.4%), each with more than 30 publications. Multiple country publications (MCP) refers to the number of articles coauthored by authors from different countries/regions, while single country publications refer to the number of articles whose authors all come from the same country/region. The proportion of MCPs can reflect the current state of international cooperation and academic research exchange in this field. From Figure 3A and Table 1, it can be observed that the USA and France, in the field of wrist arthroscopy, have published over 10 articles in collaboration with other countries/regions, indicating more abundant academic exchanges. In contrast, other countries exhibit relatively fewer international collaborations and academic exchanges. From the ranking of countries with the most citations in Figure 3B, it can be seen that the USA is in an absolute leading position with 1353 citations, demonstrating its authority in the field of wrist arthroscopy.

**Table 1:** Top 10 corresponding author’s countries.

Rank	Country	Articles	SCP	MCP	Centrality
1	USA	149	125	24	0.43
2	France	65	49	16	0.13
3	China	62	56	6	0.09
4	Germany	42	35	7	0.11
5	Japan	36	32	4	0.06
6	Korea	35	34	1	0
7	United Kingdom	34	29	5	0.22
8	Netherlands	23	17	6	0
9	Australia	20	13	7	0.08
10	Spain	18	14	4	0.3

MCP = multiple country publications, SCP = single country publications.

From Figure 3C and D, and Table 2 of the countries’ collaboration network, further information about the cooperation between the USA, France, and other countries is revealed. The USA collaborates with Australia and China, while France collaborates with Belgium and Japan, each having coauthored more than 6 papers. Additionally, China, Germany, the United Kingdom, and Spain have also carried out collaborations with different countries/regions, forming 6 clusters. The Countries’ Collaboration World Map in Figure 3E shows that there are some connections between countries, but they are sparse and scattered, corresponding to the fact that single country publications far outnumber multicountry publications. This indicates that the majority of academic research in this field is conducted locally by most countries/regions. Although there is a certain foundation of international cooperation, further strengthening cooperation among countries is still needed.

**Table 2:** Top 10 countries in terms of cooperation quantity.

Rank	From	To	Frequency
1	USA	Australia	7
2	France	Belgium	7
3	France	Japan	6
4	USA	China	6
5	USA	France	6
6	China	United Kingdom	5
7	USA	Germany	5
8	USA	Japan	5
9	USA	Netherlands	5
10	France	Netherlands	4

### 3.3. Analysis of institutions

**Figure 4.**
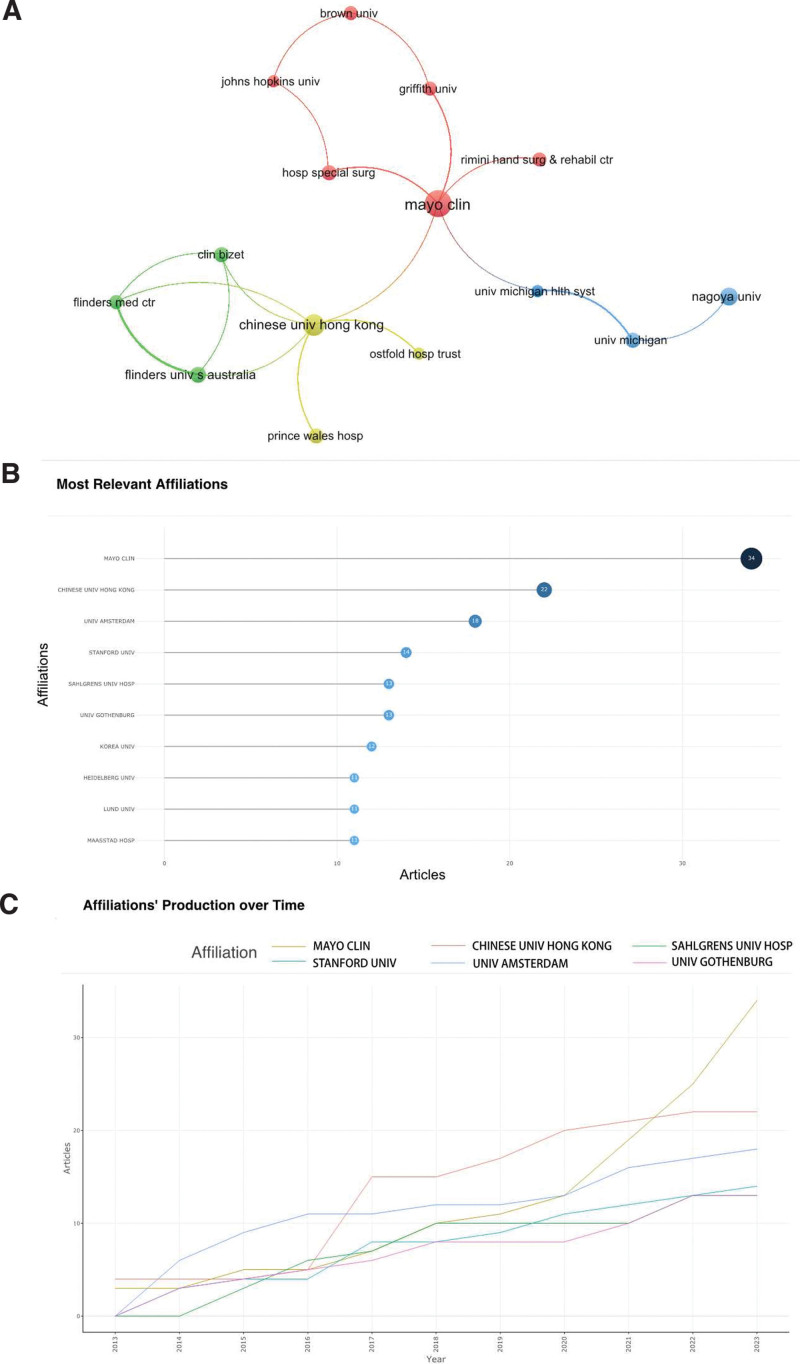
Institutional status of wrist arthroscopy publications. (A) The visualization of institutions cooperation networks. (B) Top 10 institutions by the number of publications. (C) Top 5 institutions production over time (Sahlgrens Univ Hosp and Univ Gothenburg are tied for fifth place).

Figure 4 and Table 3 depict the publishing landscape of institutions engaged in wrist arthroscopy research. Notably, among the top 10 institutions publishing the most on wrist arthroscopy, the Mayo Clinic leads with the highest number of publications (N = 34), trailed by the Chinese University of Hong Kong (N = 22), and the University of Amsterdam (N = 18). It’s worth mentioning that each institution within this top 10 list has published more than 10 publications. Specifically, the details of the publication counts and rankings of these institutions are displayed in Figure 4B.

**Table 3:** Top 10 institutions for wrist arthroscopy research.

Rank	Affiliation	Articles	Citations
1	Mayo Clin	34	106
2	Chinese Univ Hong Kong	22	213
3	Univ Amsterdam	18	69
4	Stanford Univ	14	60
5	Sahlgrens Univ Hosp	13	110
6	Univ Gothenburg	13	139
7	Korea Univ	12	59
8	Heidelberg Univ	11	145
9	Lund Univ	11	50
10	Maasstad Hosp	11	37

The “Affiliations’ Production over Time” for the top 6 publishing institutions is illustrated in Figure 4C. Here, the Mayo Clinic stands out, having experienced the fastest growth rate. This institution started its explosive growth in 2020 and managed to surpass the Chinese University of Hong Kong by 2021 to clinch the top spot. Meanwhile, the Chinese University of Hong Kong kick-started its rapid increase in publications from 2016 and has since sustained high output levels.

Figure 4A provides a visualization of the institution collaboration network. The publishing institutions in the field of wrist arthroscopy are primarily divided into 4 clusters. The collaboration hub among these institutions orbits around the Mayo Clinic and the Chinese University of Hong Kong, which indicates that the above 2 agencies have a core position in the field of wrist joint. Despite the University of Amsterdam’s notable third place ranking in terms of academic output in this field, it does not frequently collaborate with other affiliated institutions, resulting in its exclusion from the depicted network.

### 3.4. Analysis of journals

#### 3.4.1. Volume of journal publications.

**Figure 5.**
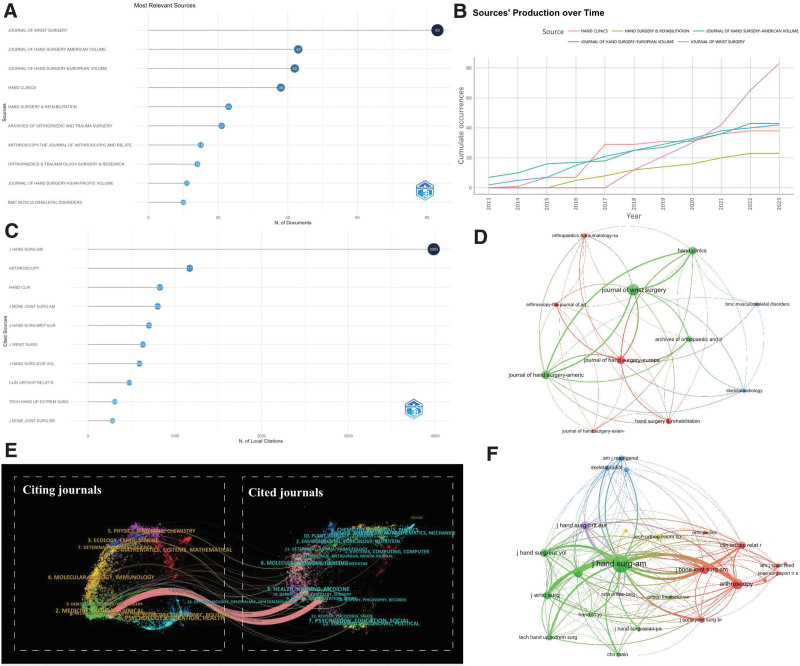
Journal status of wrist arthroscopy publications. (A) Top 10 journals by the number of publications. (B) Top 5 journals production over time. (C) Top 10 most local cited journals. (D) Network map of journals that were cited in more than 10 citations. (E) The dual-map overlay of journals related to wrist arthroscopy. (F) Network map of journals that were co-cited in more than 100 citations.

As illustrated in Figure 5A, the top 10 journals are listed in terms of the total number of publications. The 5 leading journals are *Journal of Wrist Surgery* (N = 83), *Journal of Hand Surgery-American Volume* (N = 43), *Journal of Hand Surgery-European Volume* (N = 42), *Hand Clinics* (N = 38), and *Hand Surgery & Rehabilitation* (N = 23). Collectively, these 5 journals account for approximately 36.06% of the total publications. This information, based on Bradford’s law, identifies these 5 journals as core sources. Nevertheless, considering the journal citation reports (JCR) partitions and impact factors, all these 5 journals are categorized into the 3rd or 4th quartiles, with impact factors ranging between 0.7 and 2.2, indicating that there is substantial room for improvement in the overall standard of journals publishing wrist arthroscopy-related works.

Figure 5B illustrates the “Sources’ Production over Time” trend for these top 5 publishing journals. *Journal of Wrist Surgery*, despite having a late start in 2017, has demonstrated an impressive trajectory. With explosive growth, it became the leading journal in publication volume by 2020, a position it continues to hold. *Journal of Hand Surgery-American Volume* and *Journal of Hand Surgery-European Volume*, as pioneers in wrist arthroscopy publications, have consistently maintained high publishing standards. Hand Clinics saw a sharp rise in its publication volume in 2016 and has been stable since. Importantly, from 2017 onward, there has been a noticeable annual increase in the volume of wrist arthroscopy-related publications in each of these journals, signaling a growing interest in this field.

#### 3.4.2. Journal citations and co-citations.

Figure 5F presents a collaborative network diagram of journals that have published over 10 wrist arthroscopy papers. The network comprises 11 journals in total, featuring *Journal of Wrist Surgery, Journal of Hand Surgery-European Volume*, and *Skeletal Radiology* as central nodes. This network is primarily divided into 3 clusters, demonstrating close collaboration among various journals. Within these 11 journals, except for *J Hand Surg-Asian-Pa*, the remaining 10 journals correspond to the top 10 journals based on citation frequency. The specific citation count ranking can be referred to in Table 4. Leading the pack is *J Hand Surg-Am* with the highest number of citations (N = 435), followed by *J Hand Surg-Eur* (N = 428), and *Arch Orthop Traum Su* (N = 324). Figure 5C and D showcases the collaborative network diagrams of the top 10 co-cited journals and journals with over 100 co-citations. The top 5 journals in this regard are: *J Hand Surg-Am* (N = 3983), *Arthroscopy* (N = 1171), *Hand Clin* (N = 827), *J Bone Joint Surg Am* (N = 802), and *J Hand Surg-Brit Eur* (N = 702). A total of 25 journals have been co-cited more than 100 times and are divided into 5 clusters, with *J Hand Surg-Am* at the core.

**Table 4:** Top 10 journals and co-citations journals of wrist arthroscopy.

Rank	Journals	Articles	Citations	IF	*Q*	Rank	Journals	Co-citations	IF	*Q*
1	*Journal of Wrist Surgery*	83	162	0.7	/	1	*J Hand Surg-Am*	3983	1.9	3
2	*J Hand Surg-Am*	43	435	1.9	3	2	*Arthroscopy*	1171	4.7	1
3	*J Hand Surg-Eur*	42	428	2.2	3	3	*Hand Clin*	827	1.1	4
4	*Hand Clinics*	38	309	1.1	4	4	*J Bone Joint Surg Am*	802	5.3	1
5	*Hand Surgery & Rehabilitation*	23	103	1.1	4	5	*J Hand Surg-Brit*	702	/	/
6	*Arch Orthop Traum Su*	21	324	2.3	3	6	*J Wrist Surg*	632	0.7	/
7	*Arthroscopy*	15	180	4.7	1	7	*J Hand Surg-Eur*	592	2.2	3
8	*Orthop Traumatol-Sur*	14	122	2.3	2	8	*Clin Orthop Relat R*	474	4.2	1
9	*J Hand Surg-Asian-Pa*	11	6	0.5	/	9	*Tech Hand Up Extrem Surg*	308	/	/
10	*BMC Musculoskeletal Disorders*	10	208	2.3	2	10	*J Bone Joint Surg Br*	282	/	/

IF = Impact Factor.

CiteSpace was utilized to display a dual map overlay of journals related to wrist arthroscopy, as demonstrated in Figure 5E. The cluster on the left side of the pink line signifies the journals citing the articles, while the cluster on the right side of the pink path represents the journals being cited. It is primarily seen that articles published under the domains of health/nursing/medicine and sports/rehabilitation/sport are most often cited by researchers in medicine/medical/clinical journals. The double map overlay of journals could suggest that the primary focus of current wrist arthroscopy research is clinical rehabilitation, particularly aiming at the restoration of motor function and postoperative care.

### 3.5. Analysis of authors

**Figure 6.**
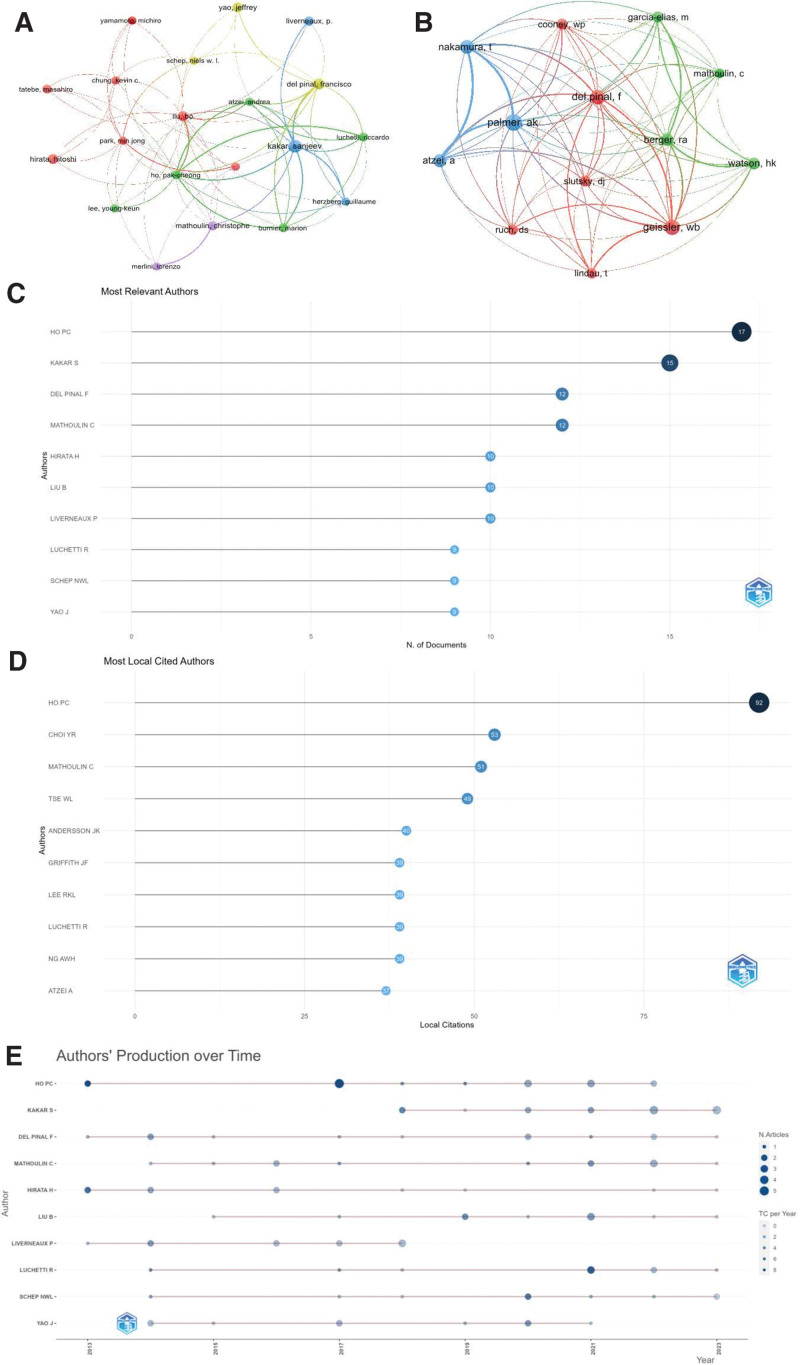
Author status of wrist arthroscopy publications. (A) Network map of authors that were cited in more than 6 citations. (B) Network map of authors that were co-cited in more than 80 citations. (C) Top 10 authors by the number of publications. (D) Top 10 most local cited authors. (E) Top 10 authors’ production over time.

Figure 6 and Table 5 display the situation of the authors of wrist arthroscopy publications. Figure 6A depicts the collaboration among authors who have published over 6 papers in the field of wrist arthroscopy research. There are 20 authors in total, primarily segmented into 5 clusters, with Kakar S (USA), Liu B (China), Ho PC (Hong Kong, China), Del Pinal F (Spain), and Mathoulin C (France) being the central figures. Figure 6B illustrates the network of authors whose research related to wrist arthroscopy technology has co-citation more than 80 times. The network consists of 13 authors, with Palmer AK, Geissier WB, and Berger RA, all from the USA, being the core authors.

**Table 5:** Top 10 authors and co-citations authors of wrist arthroscopy.

Rank	Authors	Articles	H-index	Country	Institutions	Rank	Authors	Citations	Total link strength	Country	Institutions
1	Ho PC	17	7	HK, China	Chinese University of Hong Kong	1	Palmer AK	232	3447	USA	SUNY Health Science Center
2	Kakar S	15	4	USA	Mayo Clinic	2	Geissler WB	205	3254	USA	University of Mississippi Medical Center
3	Del Pinal F	12	7	Spain	Hospital Mutua Montañesa	3	Del Pinal F	189	2857	Spain	Hospital Mutua Montañesa
4	Mathoulin C	12	6	France	Clinique Bizet	4	Nakamura T	162	2735	Japan	Keio University
5	Hirata H	10	5	Japan	Mie University	5	Atzei A	153	2656	Italy	Italy Hand Surgery Unit
6	Liu B	10	4	China	Beijing Ji Shui Tan Hospital	6	Berger RA	126	2580	USA	Mayo Clinic
7	Liverneaux P	10	4	France	University Hospital of Strasbourg	7	Watson HK	122	1833	USA	Hartford Hospital
8	Luchetti R	9	5	Italy	Rimini Hand and Rehabilitation Center	8	Slutsky DJ	110	1607	USA	Harbor-UCLA Medical Center
9	Schep NWL	9	4	Netherlands	Maasstad Hospital	9	Cooney WP	100	1499	USA	Mayo Clinic
10	Yao J	9	5	USA	Stanford University Medical Center	10	Garcia EM	98	1886	Spain	Kaplan Institute

UCLA = University of California at Los Angeles.

In the ranking of author publications, the top 10 authors combined have published 113 papers, approximately 17.8% of the total number of papers published in this field (Fig. 6C). Ho PC is the most productive author, having published 17 papers, followed by Kakar S with 15 papers, and Del Pinal F and Mathoulin C, each with 12 papers (Fig. 6C). Figure 6D represents the top 10 authors with the highest number of citations, with the top 5 being Ho PC, Choi YR, Mathoulin C, Tse WL, and Andersson JK. Among them, Ho PC, who is in the first place, has been cited 92 times. Ho PC, being the author with the highest number of publications as well as the highest number of citations, underscores his central role in the field of wrist arthroscopy. Figure 6E shows the timeline of the top ten authors in terms of the number of publications. Among the top 10 authors, 4 began researching wrist arthroscopy technology as early as 2013, and the number of publications began to increase significantly from 2020 to 2022. The H-index is a metric used to evaluate the productivity and citation impact of a researcher’s publications. It is defined as the maximum value of H such that the given author has published h papers that have each been cited at least H times. The H-index is intended to measure both the productivity (number of papers) and the impact (number of citations) of the researcher’s work. As shown in Table 5, Ho PC and Del Pinal F are the authors with the highest H-index, both being 7.

### 3.6. Analysis of highly cited publications and references

**Figure 7.**
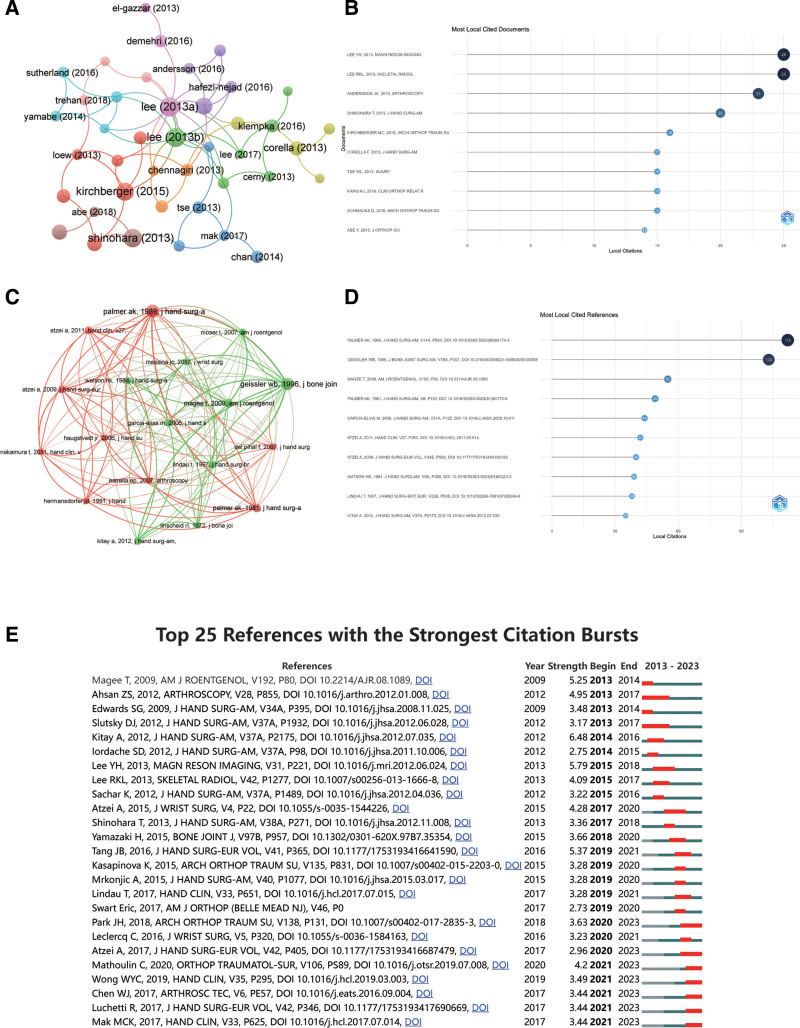
The citation and co-citation status of wrist arthroscopy publications and references. (A) Network map of publications that were cited in more than 15 citations. (B) Top 10 publications with the most citations. (C) Network map of references that were co-cited in more than 30 citations. (D) Top 10 references with the most citations. (E) Top 25 references with the strongest citation bursts.

In order to delve into the main content and progress in the field of wrist arthroscopy, we conducted an analysis of citation patterns for the relevant publications and references. Figure 7A and B presents the publications that have been cited more than 15 times among the 635 publications we incorporated, and also the top 10 most frequently cited publications. The 2 papers with the highest number of citations are “Lee YH, 2013, *Magn Reson Imaging*”^[21]^ and “Lee RKL, 2013, *Skeletal Radiol*,”^[22]^ each being cited 25 times. These papers leverage wrist arthroscopy as the gold standard for evaluating the sensitivity, specificity, and accuracy of Multidetector CT arthrography, 3-T magnetic resonance imaging (MRI), magnetic resonance arthrography, and other methodologies in diagnosing tears of the intrinsic wrist ligament and the TFCC. Through these evaluations, the pivotal role of wrist arthroscopy in diagnosing soft tissue injuries of the wrist has been indirectly accentuated.

The co-citation network of references conducted by VOSviewer (Fig. 7C) highlights 3 main clusters, each containing a large number of references. This implies extensive interconnections among these citations. As shown in Figure 7C, there is a close co-citation relationship between “Palmer AK, 1989, *J Hand Surg-A*”^[23]^ and “Palmer AK, 1981, *J Hand Surg-A*.”^[24]^ Figure 7D displays the top 10 most frequently cited references, with “Triangular fibrocartilage complex lesions: A classification”^[23]^ ranking first, accumulating 112 citations. This review looks back on clinical experiences based on anatomical and biomechanical research, proposing a classification of TFCC injuries. This classification is based on clinical examinations, conventional X-ray tilt procedures, wrist arthrography, wrist arthroscopy, and wrist arthrotomy. The classification can distinguish between traumatic lesions and degenerative alterations. The paper titled “Intracarpal soft-tissue lesions associated with an intra-articular fracture of the distal end of the radius”^[25]^ ranks second, garnering 103 citations. The article elaborates on how 60 cases with intra-articular displaced fractures at the distal end of the radius underwent manual repositioning and internal fixation under fluoroscopy and arthroscopy guidance.

Additionally, references that receive extensive citations from scholars during a certain time frame within a topic are referred to as references exhibiting citation bursts. These are valuable measurement indicators, accentuating the references that ignite interest of researchers in a specific domain within a particular time period. In this study, CiteSpace identified the top 25 references with the most robust citation bursts, as shown in Figure 7E. Among them, Magee T’s 2009 article “Comparison of 3-T MRI and arthroscopy of intrinsic wrist ligament and TFCC tears”^[26]^ ranks the highest (strength = 5.25). Next, Ahsan ZS,^[27]^ Edwards SG^[28]^ reviewed the incidence of complications related to wrist arthroscopy, the objective and subjective outcomes of prospective arthroscopic dorsum tendon cystectomy, and identified and examined intra-articular lesions associated with the tendon sheath cyst. Furthermore, the 2020 paper by Mathoulin C titled “Role of wrist arthroscopy in scapholunate dissociation”^[9]^ has been explosively cited in recent years (2021–2023). This paper reviews the anatomical underpinnings and clinical treatment of scapholunate dissociations, emphasizes the therapeutic efficacy of wrist arthroscopy for this disease, and analyzes the limitations and prospects of using wrist arthroscopy to treat this condition. The significance of this paper lies in its contributions to selecting appropriate treatment modalities for chronic scapholunate ligament injuries and in the wider applications of wrist arthroscopy.

### 3.7. Analysis of keywords

#### 3.7.1. Analysis of keyword bursts.

**Figure 8.**
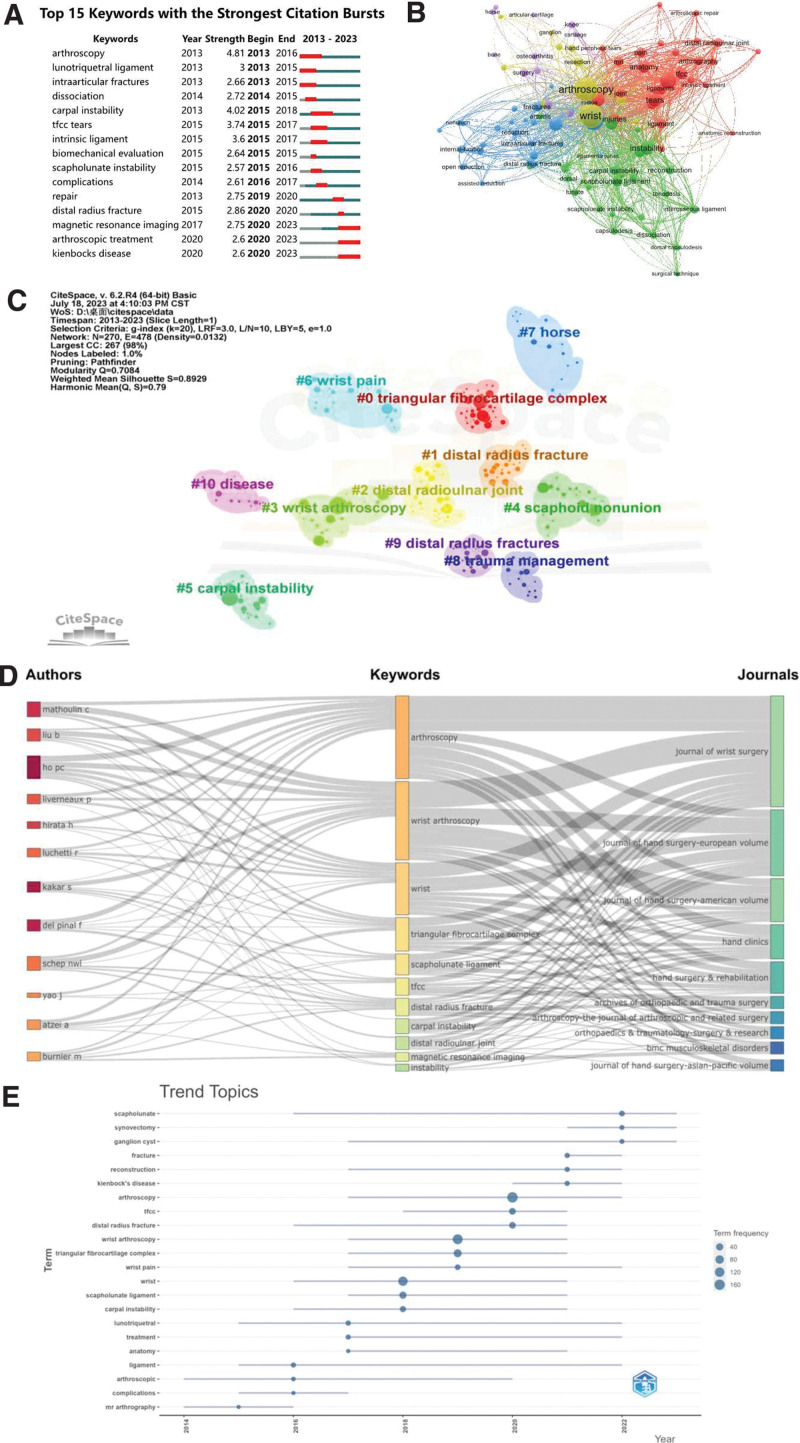
Keywords status of wrist arthroscopy publications. (A) Top 15 keywords with the strongest citation bursts. (B) Keyword network map with keywords appearing more than 10 times. (C) Keyword clustering analysis chart. (D) Three-field plot of the keywords analysis. (E) The historical migration of research hotspots.

Keyword bursts represent substantial increases in the frequency of a keyword’s appearance within a short duration. Conducting a keyword burst analysis allows us to better discern the research topics of interest during specific periods, and thereby predict the direction of research development. Figure 8A displays the top 15 keywords with the highest burst strength. The most influential keyword is “arthroscopy” (strength = 4.81), followed by “carpal instability” (4.02) and “tfcc tears” (3.74). Keywords such as “arthroscopy,” “carpal instability,” “magnetic resonance imaging,” “arthroscopic treatment,” “kienbock’s disease,” among others, exhibited lengthy burst durations, each spanning 2 years. Significantly, “kienbock’s disease” had the longest burst period and also experienced the most recent burst in citations (2020–2023), indicative of the potential for wrist arthroscopy to diagnose and treat Kienböck disease. This suggests that this topic could become a prominent future research focus and hotspot.

#### 3.7.2. Keyword co-occurrence analysis.

Out of a total of 2080 keywords, we utilized VOSviewer to generate a co-occurrence map for keywords appearing more than 10 times, comprising 102 keywords in total (Fig. 8B). The top 5 keywords in terms of frequency include “wrist arthroscopy,” “wrist,” “triangular fibrocartilage complex,” “lesions,” and “management” (Table 6). The primary focus in the field of wrist arthroscopy is the diagnosis and management of wrist-related injuries, particularly those pertaining to the TFCC. This trend underscores the significance of minimally invasive methods for diagnosing and treating wrist injuries, further suggesting that prevailing themes in wrist joint research revolve around comprehending the intricate anatomical structure of the wrist, diagnosing and classifying injuries, and studying interventions like arthroscopy for injury management.

**Table 6:** Top 20 keywords of wrist arthroscopy.

Rank	Keywords	Occurrences	Centrality
1	wrist arthroscopy	175	0.05
2	wrist	130	0.05
3	triangular fibrocartilage complex	112	0.04
4	lesions	76	0.08
5	management	76	0.09
6	tears	73	0.03
7	arthroscopy	65	0.06
8	injury	65	0.05
9	instability	60	0.06
10	diagnosis	59	0.1
11	anatomy	52	0.06
12	repair	50	0.07
13	scapholunate ligament	44	0.05
14	fractures	34	0.14
15	pain	34	0.07
16	classification	32	0.06
17	carpal instability	32	0.03
18	joint	30	0.07
19	arthrography	27	0.11
20	distal radius fracture	27	0.04

#### 3.7.3. Keyword clustering analysis.

We used CiteSpace for keyword co-occurrence clustering analysis, and keyword labels were used to extract cluster labels. The log-likelihood ratio algorithm was used for keyword clustering to obtain a clustering map. The modularity (*Q* value) of the network is a global measure of the overall structure; the higher the *Q* value, the better the clustering. *Q* > 0.3 indicates that the clustering structure is significant. The average silhouette value (*S* value) is a measure used to assess network homogeneity, a value close to 1 indicates higher homogeneity. The keyword clustering analysis included in this study is shown in Figure 8C, with a *Q* value of 0.79 and an *S* value of 0.8929, indicating that this clustering is significant and highly reliable. We obtained a total of 11 valid clusters, with the top 5 being #0 TFCC, #1 distal radius fracture, #2 distal radioulnar joint, #3 wrist arthroscopy, #4 scaphoid nonunion. This indicates that the main focus of relevant scholars is in the treatment of common wrist diseases such as TFCC injuries and distal radius fractures through wrist arthroscopy.

#### 3.7.4. Three-field plot analysis.

Figure 8D shows a 3-field plot of authors, keywords, and publishing journals. This plot can help us better comprehend the relationship between them, where a thicker line denotes a higher frequency. The most frequently used keywords are “arthoscopy,” “wrist arthroscopy,” “wrist,” “triangular fibrocartilage complex,” and “scapholunate ligament.” Authors Mathoulin C, Ho PC, Liu B are closely related to the keywords “arthoscopy,” “wrist arthroscopy,” “wrist,” establishing the strongest connections. Simultaneously, the strongest connections are most relevant to the 3 journals: *Journal of Wrist Surgery, Journal of Hand Surgery-American Volume*, and *Journal of Hand Surgery-European Volume*. This shows that these 3 journals encompass most publications related to the aforementioned keywords.

#### 3.7.5. Keyword timeline analysis.

By analyzing the timeline distribution of keywords, we can better understand the historical changes in the research hotspots in this field and forecast future research trends. We displayed some popular keywords in bibliometrix over the past decade, with the word minimum frequency set at 5 and the number of words per year at 3. As illustrated in Figure 8E, the result includes a total of 22 keywords and simultaneously displays their frequencies and periods of popularity. Over the past decade, we can see that the research focus in the field of wrist arthroscopy has shifted from more fundamental anatomical knowledge, such as “ligament,” to more complex diagnostic and treatment techniques like “magnetic resonance arthrography” and “arthroscopic.” This could be attributed to advancements in technology, which enable doctors to diagnose and treat orthopedic diseases with greater precision. Then we see the appearance of “wrist” and related structures (such as “scapholunate ligament” and “triangular fibrocartilage complex”), which may indicate that researchers are paying more attention to specific anatomical structures and diseases during this period. This may be due to an improved understanding of the anatomy and function of specific areas, as well as an increased demand for the treatment of diseases in these areas. In the past 3 years, we have observed the emergence of “fracture,” “reconstruction,” and specific diseases (like “kienbock’s disease”). This might suggest that the focus of research has shifted from anatomy and fundamental diseases to specific clinical treatments and surgical techniques. This could be due to the advancements in science and technology, allowing doctors to manage more complex surgical procedures and treatment plans. Considering these trends, future research may continue to focus on specific diseases and treatment plans, but it might incorporate more technical elements, such as the application of artificial intelligence and machine learning in diagnosis and treatment.

## 4. Discussion

### 4.1. Progress in wrist arthroscopy studies

The wrist joint is a complex structure composed of multiple bones, ligaments, and tendons that work together to allow us to use our wrists flexibly for various activities.^[29]^ However, due to the complexity of this region, diagnosing the cause of wrist pain has always been a complicated and challenging issue; any damage or disease in any part can lead to pain.^[30]^ Additionally, various ailments can manifest with similar symptoms, further complicating a clear diagnosis. Many wrist disorders, such as scapholunate interosseous ligament injury and osteonecrosis of the lunate, often do not show positive findings in early X-rays, and even more detailed CT scans might not identify the issue. Even MRI scans of the wrist often struggle to make a definitive diagnosis.^[31–33]^ Although wrist arthrography provides some indirect signs for disease diagnosis to a certain extent, there’s still a notable false positive and false negative rate associated with this method, reducing the reliability of this diagnostic method.^[34–36]^ Thus, there’s a pressing need to find more precise and intuitive diagnostic tools to elevate the diagnostic accuracy for wrist pain.

The invention of wrist arthroscopy is undoubtedly a milestone in the field of hand surgery. Since its first report in the early 1970s,^[37]^ it has evolved into a crucial tool for diagnosing and treating various wrist conditions. Initially, this technology was mainly used for diagnosing wrist disorders that could not be identified using conventional methods, such as tears of the TFCC and intercarpal ligament injuries.^[38]^ As surgical techniques advanced and surgical tools improved, the design and capabilities of wrist arthroscopy continually refined. It has broadened its scope to treat various wrist afflictions, such as wrist fractures and instability.^[39–41]^ This specialized technique has enabled physicians to perform surgeries through smaller incisions. This minimally invasive method not only diminishes surgical trauma but also accelerates the patient’s recovery period.^[42]^ Furthermore, the integration of precise surgical instruments and high-definition imaging has rendered complex surgeries feasible, like mending injured ligaments or excising diseased tissues.^[3]^ The enhancements in wrist arthroscopy technology have facilitated significant advancements in its surgical application.

So far, wrist arthroscopy has become an essential tool for managing various wrist diseases. Whether it’s common wrist arthritis, complex distal radioulnar joint instability, or intricate procedures needed for TFCC injuries, wrist arthroscopy offers effective diagnosis and treatment methods.^[43–45]^ Additionally, wrist arthroscopy can also be used to assess surgical outcomes and conduct postoperative reviews, further enhancing the precision and efficacy of the treatment. While wrist arthroscopy technology has made significant strides, there are still many challenges in this field. For example, improve the accuracy of the surgery to ensure treatment outcomes, how to reduce complications such as neurovascular injuries, and how to accelerate the patient’s recovery speed, etc.^[46,47]^ These issues require further exploration and resolution in our future research. Meanwhile, we also look forward to the emergence of new technologies and equipment that can further advance wrist arthroscopy technology, enhancing its role in the diagnosis and treatment of wrist diseases.

In recent years, with the extensive application of wrist arthroscopy technology in practice, the number of related research papers has also been on the rise. Yet, even with numerous studies and publications in this realm, our grasp on its overarching research trends, focal points, and international collaborations remains scant. The dearth of holistic and unbiased information poses challenges for doctors, researchers, and policymakers when making decisions. Therefore, by delving into the development trends and research hotspots of wrist arthroscopy technology over the past decade, we can better understand its evolution, identify potential research opportunities, and anticipate future challenges. This endeavor won’t just facilitate a better assessment of wrist arthroscopy technology’s progression but will also be instrumental in guiding our future research in this area. Based on this, this study employs tools like VOSviewer, Citespace, the R package “bibliometrix,” and online analysis platforms. Utilizing intricate bibliometric principles and advanced visualization techniques, we analyze publications related to wrist arthroscopy in the Web of Science Core Collection database, unveiling the primary research hotspots and trends.

### 4.2. Analysis of results

Through analyzing the outcomes of this research, we can deduce the research focal points of wrist arthroscopy and anticipate upcoming trends. First and foremost, it’s evident that research on wrist arthroscopy techniques is actively underway worldwide. An increasing number of publications indicate that researchers are constantly seeking advancements and improvements in wrist arthroscopy surgery, instruments, and techniques. As a minimally invasive surgical method, wrist arthroscopy offers several advantages, such as reduced postoperative pain, faster recovery time, and shorter hospital stays. The escalating research enthusiasm in this domain indicates the drive to further enhance these advantages, and there’s potential to broaden wrist arthroscopy applications to an expansive array of wrist-linked ailments. Additionally, the positive research trend highlights the ongoing collaboration and knowledge-sharing among researchers, surgeons, and medical professionals in this field. This collaboration helps disseminate best practices and formulate evidence-based guidelines for optimal use of wrist arthroscopy in a clinical setting. Persistent enthusiasm in this realm might stimulate more investment in research and development, spawning innovative surgical instruments, pioneering imaging techniques, and state-of-the-art approaches to tackle intricate wrist disorders. Such progressions might bolster the efficacy and safety levels of wrist arthroscopy evaluations, rendering it an ever-essential instrument for orthopedists and wrist experts.

In the past decade, wrist arthroscopy technology has garnered widespread attention and development globally, especially in the USA, France, and China. In this field, the USA undoubtedly leads, holding the greatest influence, both in terms of the number of papers published and the number of citations. This might be attributed to their robust medical research capabilities and their proactive adoption and promotion of new technologies. In terms of MCP, the USA and France have demonstrated a relatively high willingness and capacity for collaboration, indicating that these 2 countries are very active in international academic exchanges in the wrist arthroscopy field. However, other countries/regions have less collaboration in this regard, possibly due to constraints in research resources, funding, or academic exchanges. From the perspective of national collaboration networks, while certain countries like the USA, France, China, and Germany have strong collaborative ties with other nations, overall, inter-country collaborations remain sparse. This implies that research in the wrist arthroscopy field is often conducted by individual countries/regions rather than based on extensive international collaborations. While the foundation for international cooperation exists, there’s significant room and potential to deepen and expand these collaborations, fostering greater exchange, and sharing. Examining the publication institutions for wrist arthroscopy, the Mayo Clinic and the Chinese University of Hong Kong stand out as frontrunners in research contributions, and they evidently play central roles within institutional collaboration networks, emphasizing their pivotal stature in the wrist arthroscopy sector. Their studies possibly set vital trajectories and benchmarks, enticing collaborations from other institutions. The Mayo Clinic, renowned as an international apex for medical clinics and research, witnessed a marked surge in its publications since 2020. This underscores the institution’s heightened resource allocation and focus on wrist arthroscopy, earmarking it as a pivotal research avenue for the future. The Chinese University of Hong Kong, on the other hand, has established a research advantage in the wrist arthroscopy field since 2016 and has consistently maintained this lead. The institution’s consistent research deliverables might correlate with its enduring research strategies, collaborative partnerships, or dedicated research groups.

It can be discerned from the journals in the wrist arthroscopy field that 5 journals, including *Journal of Wrist Surgery, Journal of Hand Surgery-American Volume*, and *Journal of Hand Surgery-European Volume*, hold central positions in the wrist arthroscopy domain, Their publication volume constitutes a major portion of the field’s literature, but their JCR categorization and impact factors are relatively low. This could suggest that the research field of wrist arthroscopy remains a specialized sector that hasn’t garnered broad interest yet. This situation perhaps reflects that wrist arthroscopy technology is still in its early stages of development, despite its obvious technical advantages and clinical application potential, it awaits further promotion and use. Further analyzing the development trends of the journals, *Journal of Wrist Surgery* started relatively later, but its publication volume exploded after 2017, quickly surpassing other journals. As a journal dedicated to wrist-related topics, this rapid growth trend might indicate that wrist arthroscopy technology has garnered increasing interest and attention from orthopedic surgeons since 2017. Another interesting observation is that even though some journals, such as *J Hand Surg-Am*, have notably high citation rates, this isn’t mirrored in their JCR segmentation and impact factors. This might suggest that while these journals’ published studies bear substantial academic significance within the domain, their influence in the wider medical community remains restricted.

Regarding the authors, the 5 main research cluster centers each have their distinct key contributors, with Kakar S, Liu B, Ho PC, Del Pinal F, and Mathoulin C recognized as the core researchers in this domain, their collaboration networks across various nations and cultural contexts, underscoring the global character of wrist arthroscopy research. Specifically, Ho PC stands out significantly in this field, not only does he lead in publication volume, but he also has the most citations, signifying his leadership in the wrist domain. Professor Ho PC is the chairman of the Asia-Pacific Wrist Association and the head of hand surgery at Prince of Wales Hospital in Hong Kong, having pioneered multiple wrist arthroscopy techniques widely applied in clinical settings. In co-citation instances, Palmer AK, Geissier WB, and Berger RA emerged as central figures, and all 3 hail from the USA, indicating the US’s leading position in this research area. The H-index, as a measure of a researcher’s productivity and influence, further underscores the significance of Ho PC and Del Pinal F. Their research mainly focuses on the application of wrist arthroscopy techniques and the diagnosis and treatment of diseases.

In terms of highly cited publications, the 2 most frequently cited articles both evaluate the wrist arthroscopy as a “gold standard” technique, highlighting its central and irreplaceable role in diagnosing soft tissue injuries of the wrist. Both articles evaluated the relative sensitivity, specificity, and accuracy of wrist arthroscopy in comparison to other imaging methods, confirming its superior position in this domain. Among frequently cited references, “Triangular fibrocartilage complex lesions: A classification”^[23]^ received the most citations. The document introduced a classification system for TFCC injuries. This categorization offers both an organized academic framework and a meaningful tool for clinical use, likely explaining its frequent citations. The second-ranked article further established the value of wrist arthroscopy technology in the diagnosis and treatment of intra-articular fractures of the distal radius. References with citation bursts are key knowledge nodes in the field, marking turning points or significant advancements in academia. Magee T’s 2009^[26]^ article, comparing with other imaging techniques, highlighted the superiority of wrist arthroscopy. In contrast, Mathoulin C’s 2020^[9]^ article delved deeply into the treatment methods for chronic scapholunate ligament injuries, especially the efficacy and limitations of wrist arthroscopy, providing guidance for subsequent research and practice.

Analyzing the keywords, the burgeoning rise of “arthroscopy” indicates a growing interest in minimally invasive surgical procedures over the past few years. Given that minimally invasive surgeries often involve smaller incisions, fewer complications, and faster recovery, they potentially enhance patient treatment results and their quality of life. “Kienbock’s disease,” a specific wrist condition, has a strong association with “arthroscopy,” which might indicate that wrist arthroscopy in the diagnosis and treatment of this disease is becoming or about to become a research focus.^[48]^ The core focus in this field, especially injuries related to the TFCC, implies the complexity of wrist anatomy. As our understanding of wrist anatomy and function deepens, diagnostic and treatment strategies might have shifted to more intricate and challenging injuries. However, the appearance of keywords such as “minimally invasive,” “complications,” and “recovery” also reveals some new trends and challenges in current research. While minimally invasive surgery can reduce patient discomfort and speed up recovery through smaller incisions, it also introduces new challenges, such as how to avoid complications and ensure surgical precision.^[49,50]^ To address these issues, current research is seeking better solutions, such as improving surgical techniques and instruments to enhance surgical accuracy and safety, and researching and developing new treatment protocols to improve surgical outcomes and patient recovery speed. Moreover, from a timeline analysis of keywords, we can observe a clear shift in the field of wrist arthroscopy from focusing on basic anatomical knowledge to more complex diagnostic and therapeutic techniques. This shift might reflect a general trend in the medical community, where, with the advancement of technology, the focus has moved from basic science to clinical applications. Looking forward, with the expansive adoption of technologies like artificial intelligence and machine learning in medicine, research in wrist-related areas will increasingly converge on these cutting-edge technologies’ applications, aiming for pinpoint diagnoses and optimized treatment plans.

### 4.3. Limitations and future research trend predictions

From the preceding analysis, we identify several limitations in wrist arthroscopy research: Given the novelty of wrist arthroscopy technology, numerous studies might be at a preliminary or observational phase. This could result in certain research findings being inconsistent or not reliable. Due to the lack of standardized guidelines, different doctors might employ varying surgical techniques, and treatment methods, leading to inconsistent treatment outcomes for patients. Consequently, training and educating newcomers become challenging, potentially leading to a deviation in the transmission of surgical techniques. Present collaborations between countries and institutions in the field of wrist arthroscopy still need to be strengthened.

Based on these observations, we foresee the following directions for future wrist arthroscopy research: To demonstrate the efficacy and superiority of wrist arthroscopy, upcoming research should focus on large-scale, multicenter randomized controlled studies. As this technique is promoted globally, it’s imperative to establish and disseminate unified techniques and treatment standards to ensure consistent and optimal patient outcomes. Strengthen inter-country and inter-institutional collaborations, jointly innovating and advancing new techniques to rapidly push the boundaries of technology.

## 5. Conclusion

Wrist arthroscopy, as a minimally invasive technique widely used in hand surgery in recent years, has demonstrated great potential in the treatment of various wrist diseases. Utilizing bibliometric analysis, we have delved into the global research landscape, focal points, and emerging trends of this technology. While wrist arthroscopy has made significant progress in treating wrist diseases like TFCC injuries and avascular necrosis of the lunate, challenges still exist in its research domain. Nonetheless, with continuous deepened research, reinforced international synergies, and constant technological evolution, wrist arthroscopy holds the potential to standardize as a primary treatment in hand surgery. This would offer more efficient and secure therapeutic choices for patients globally, simultaneously advancing the broader medical domain.

## Author contributions

**Writing—original draft:** Chengyin Lu.

**Data curation:** Zhiqiang Luo, Li Zeng, Zehua Rao, Mingxuan Wang.

**Writing—review & editing:** Xiaohui Wang, Hui Xiong, Biao Zhou.
